# Deep learning for acute rib fracture detection in CT data: a systematic review and meta-analysis

**DOI:** 10.1093/bjr/tqae014

**Published:** 2024-01-13

**Authors:** Manel Lopez-Melia, Virginie Magnin, Stéphane Marchand-Maillet, Silke Grabherr

**Affiliations:** University Centre of Legal Medicine Lausanne-Geneva, Geneva 1206, Switzerland; University Hospital and University of Geneva, Geneva 1205, Switzerland; University Centre of Legal Medicine Lausanne-Geneva, Geneva 1206, Switzerland; University Hospital and University of Geneva, Geneva 1205, Switzerland; University Hospital and University of Lausanne, Lausanne 1005, Switzerland; Department of Computer Science, Viper Group, University of Geneva, Carouge 1227, Switzerland; University Centre of Legal Medicine Lausanne-Geneva, Geneva 1206, Switzerland; University Hospital and University of Geneva, Geneva 1205, Switzerland; University Hospital and University of Lausanne, Lausanne 1005, Switzerland

**Keywords:** rib fracture, CT, computed tomography, deep learning

## Abstract

**Objectives:**

To review studies on deep learning (DL) models for classification, detection, and segmentation of rib fractures in CT data, to determine their risk of bias (ROB), and to analyse the performance of acute rib fracture detection models.

**Methods:**

Research articles written in English were retrieved from PubMed, Embase, and Web of Science in April 2023. A study was only included if a DL model was used to classify, detect, or segment rib fractures, and only if the model was trained with CT data from humans. For the ROB assessment, the Quality Assessment of Diagnostic Accuracy Studies tool was used. The performance of acute rib fracture detection models was meta-analysed with forest plots.

**Results:**

A total of 27 studies were selected. About 75% of the studies have ROB by not reporting the patient selection criteria, including control patients or using 5-mm slice thickness CT scans. The sensitivity, precision, and F1-score of the subgroup of low ROB studies were 89.60% (95%CI, 86.31%-92.90%), 84.89% (95%CI, 81.59%-88.18%), and 86.66% (95%CI, 84.62%-88.71%), respectively. The ROB subgroup differences test for the F1-score led to a *p*-value below 0.1.

**Conclusion:**

ROB in studies mostly stems from an inappropriate patient and data selection. The studies with low ROB have better F1-score in acute rib fracture detection using DL models.

**Advances in knowledge:**

This systematic review will be a reference to the taxonomy of the current status of rib fracture detection with DL models, and upcoming studies will benefit from our data extraction, our ROB assessment, and our meta-analysis.

## Introduction

Rib fractures are the most common injury in blunt chest trauma patients.^1^ Although a chest radiograph may suffice to diagnose displaced fractures, a multidetector CT (MDCT, or CT for simplicity) scan is recommended to ensure a more sensitive report. However, due to the complexity of CT scans, between 25% and 35% of non-displaced rib fractures are missed in diagnoses.^2^

Deep learning (DL) models, and other artificial intelligence models, can increase the diagnostic accuracy, reduce inter-reader variability, and shorten reading time.^3^^,^^4^ DL models consist of artificial neural networks with multiple layers to capture different levels of abstraction from the data. A particular configuration of neural networks is a convolutional neural network, which is powerful and efficient in computer vision thanks to the use of small kernels of parameters to capture local features.^5^

DL models have been used for the analysis of medical imaging in three main applications: classification (eg, benign vs malignant lesion), detection (eg, lesion localization), and segmentation (eg, organ contouring).^6^ In particular, DL models have been used in many studies for the detection of orthopaedic fractures, including rib fractures, with an accuracy close to that of experienced radiologists.^7^ Similarly, DL models have been applied to the analysis of postmortem CT (PMCT) scans to perform tasks such as automatic segmentation of organs, identification of mass disaster victims, or sex and age estimation in the investigation of unknown remains.^8^

Despite their promising results, DL models still face several challenges due to their strong dependence with data quantity and quality, not to mention their low interpretability.^9^^,^^10^

The objective of this systematic review is to study rib fracture classification, detection, and segmentation in CT data with DL models, both in clinical and in postmortem (PM) cases. In addition, the risk of bias (ROB) and the concerns about applicability (CAA) of the selected studies are assessed, and the impact of ROB on acute rib fracture detection performance is analysed. The review will be useful as a reference for radiologists and future research projects.

## Methods

This systematic review was registered in the PROSPERO international prospective register of systematic reviews, and it followed the guidelines proposed by the Preferred Reporting Items for Systematic reviews and Meta-Analyses (PRISMA) 2020 statement.^11^

All steps of the methods were conducted by the first author, who has one year of research experience in artificial intelligence for medical imaging. For the ROB and CAA assessment, articles were further reviewed to balance the answers to each signalling question.

### Literature search

Articles were retrieved between the 3rd and 5th of April 2023 from PubMed, Embase, and Web of Science using the query of keywords *((deep learning) OR (convolutional network)) AND (rib fracture)*.

Only the studies falling into the following criteria were included in the systematic review: (1) written in English, (2) published as a journal article or as a conference paper, (3) used a DL model, (4) the DL model was used to classify, detect, or segment rib fractures, (5) the DL model was trained on data coming from CT scans, and (6) the CT scans were taken from humans.

Studies using PMCT scans to train their models were also considered. Moreover, studies with patients with healing and old rib fractures were also included in the systematic review.

### Data extraction

Study characteristics and model performance metrics were extracted without using automation tools. To complete unreported data, corresponding authors were contacted twice within three weeks. All data were collected in Excel spreadsheets.

For classification and detection models, the extracted performance metrics were the sensitivity (or recall), the precision (or positive predictive value), and the F1-score, all at lesion level (except for two studies on rib fracture classification, which only reported performance at scan level). For segmentation models, the extracted performance metrics were the Dice score and the intersection over union. See the Supplementary Material for the formulae of these performance metrics.

In studies comparing the performance of different models on the same testing dataset, only the model with best performance was considered.

### Risk of bias assessment

The Quality Assessment of Diagnostic Accuracy Studies^12^ tool was used to assess the ROB and the CAA of the included studies. The suggested list of signalling questions was modified to adapt it to the objectives of this systematic review. See the Supplementary Material for the full list of signalling questions used in each domain.

For each study, each signalling question was answered and classified into one of the following four categories, ordered by level of risk: *low*, *no information*, *some concerns,* and *high*. Then, each domain was also classified, assigning the category with the maximum level of risk of those obtained in the signalling questions in that domain. Finally, each study was given an overall assessment following the same procedure.

The results of these assessments were presented in the form of traffic light plots (in the Supplementary Material) and summary plots, all generated with the *robvis* tool.^13^

### Meta-analysis

For rib fracture detection studies, the sensitivity, the precision, and the F1-score of their models were analysed with forest plots. The purpose of these forest plots was to visualize and compare the results of the selected models and to compute a global performance of rib fracture detection with DL models. However, we remind the reader that each study used a different dataset and a different DL model to achieve the results. Therefore, we could not extract strong conclusions concerning which of these algorithms was the best suited for this task.

Only studies reporting performance metrics of acute rib fracture detection were included in the meta-analysis. That is, studies that trained the model with acute, healing, and old rib fracture annotations and only reported a global performance were excluded. In addition, only the studies providing the 95% CI of the sensitivity and the precision were selected. If the F1-score was not reported with 95% CI, it was simulated with the Monte Carlo method.

Heterogeneity was calculated with the *I*^2^ statistic and complemented with the results obtained for the variance *τ*^2^ of the random effects (RE) model, which was chosen over the fixed-effects model because the studies could not be considered to be coming from the same population.^14^

The meta-analysis was repeated with two subgroups of acute rib fracture detection studies: those judged as having a *low* ROB and those judged as having a higher level of ROB, namely *no information*, *some concern,* or *high*. The *p*-value used for significance in the subgroup differences test was 0.1.^15^

All plots and statistical analyses were performed with the R (version 4.2.3) package *metafor*^16^ (version 4.0.0). Find the data and scripts to generate the forest plots in https://github.com/manellopez13/dl4rf_meta_analysis.

## Results

### Literature search

Using the search strategy stated in the methods, a total of 132 records were retrieved from the databases. From these, 68 records were not considered because they were duplicates, and 10 were excluded because they were either not written in English, not journal articles or conference papers, or not available online. Afterwards, the reports of the remaining records were assessed, which led to the following 27 exclusions: 8 studies did not use DL, 10 records of studies did not have rib fracture classification, detection, or segmentation as their objective, and 9 studies used chest radiographs instead of CT data to train their models.

**Figure 1.**
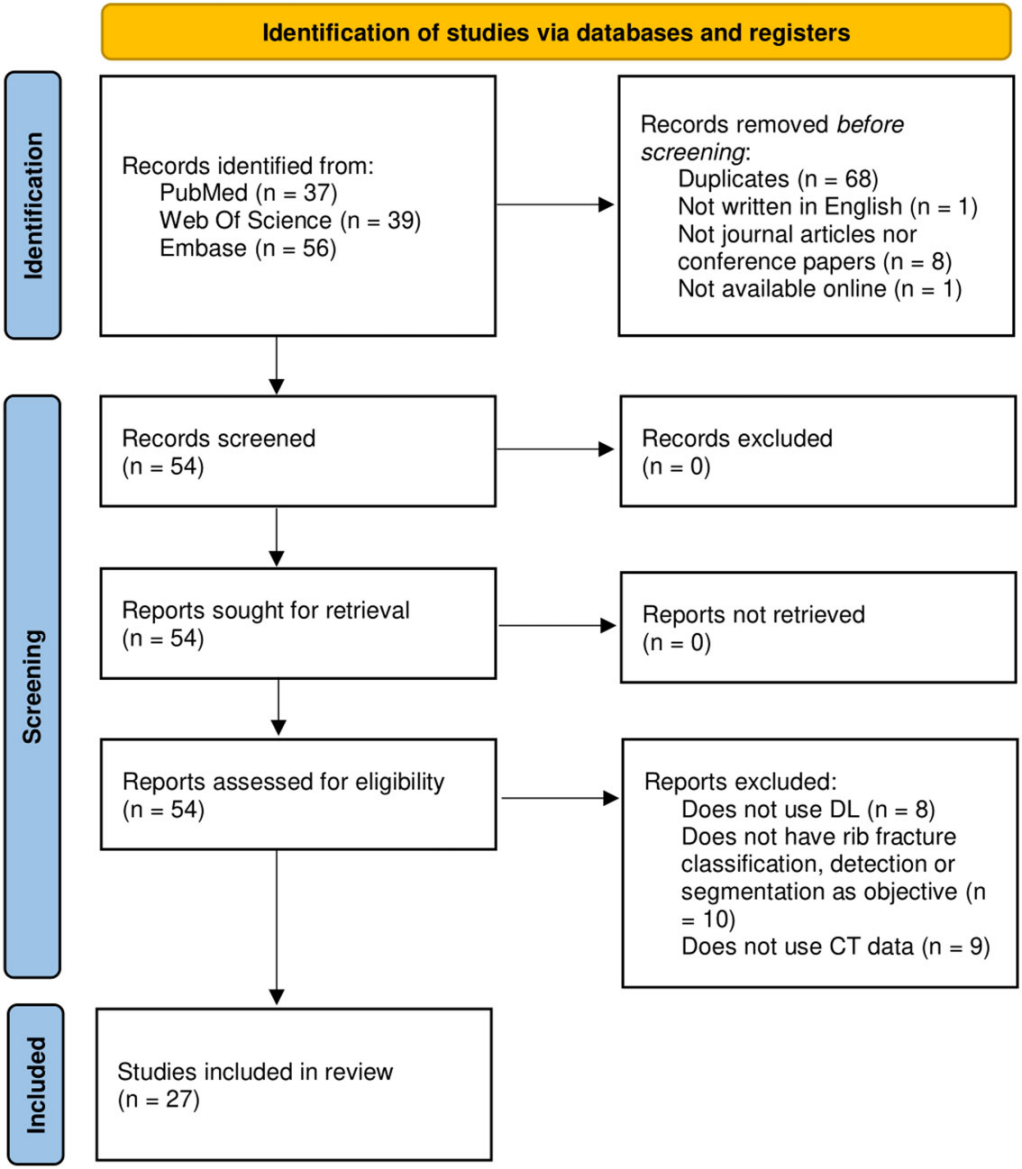
PRISMA flow diagram of included articles. DL: deep learning.

The total number of studies included in the systematic review was *n *=* *27.^17–43^ The PRISMA flow diagram of Figure 1 shows the study exclusion process.

### Data extraction

The included studies were published between 2020 and 2023. The models of three studies only classify rib fractures, and the model of one study only performed rib fracture segmentation. The remaining 23 studies trained models that detect rib fractures. Most of the models were trained with medium to high-resolution CT scans, with slice thickness ranging from 1 to 5 mm. The ratio of female patients ranged from 30% to 40% in most of the study designs, and the average age of the patients was in the range of 50-60 years. The rest of the study characteristics are gathered in Table 1. For more information on the selection criteria of the patients, refer to the Supplementary Material.

**Table 1.** Characteristics of the included studies.

Study	Reference	Type	Year	Country	Centres	CT scans	FR (%)	Age (years)	DA	CA
S1	Azuma M^17^	D	2021	Japan	1	569	30	63 (20-81)	–	–
S2	Castro-Zunti^18^	C	2021	Canada + South Korea	1	612	35	68 (17-91)	–	–
S3	Edamadaka^19^	D	2023	United States	2	475	36	55 (21-94)	–	–
S4	Gao^20^	S	2022	China	2	600	–	–	–	–
S5	Hongbiao^21^	D	2022	China	3	1623	33^a^	54 (46-64)^a^	–	–
S6	Hu^22^	C	2021	China	1	1697	–	–	Yes	Yes
S7	Ibanez^23^	C	2021	Switzerland	1	195	28	56 (8-94)	–	Req
S8	Inoue^24^	D	2022	Japan	1	200	35	54	Req	–
S9	Jin^25^	D + S	2020	China	1	900	36	55 (21-94)	Yes	Yes
S10	Kaiume^26^	D	2021	Japan	20	3683	28	58 (20-91)	–	Com^b^
S11	Li^27^	D	2023	China	7	15853	44	58	Req	–
S12	Lin^28^	D	2023	China	1	2150	50	(18-85)	–	–
S13	Meng^29^	D	2021	China	Multiple	8829	37	55	–	–
S14	Niiya^30^	D	2022	Japan	2	1045	36	58 (21-91)	Req	–
S15	Su^31^	D	2023	China	1	30	–	–	–	–
S16	Wang^32^	D	2022	China	18	13821	39	51 (18-83)	–	–
S17	Wang^33^	D + S	2023	China	1	500	36	55 (21-94)	–	–
S18	Weikert^34^	D	2019	Switzerland	9	11965	–	58	–	Com
S19	Wu^35^	D	2021	China	6	2530	32	54 (19-87)	–	–
S20	Yang^36^	D	2022	China	6	9882	54	60^c^ (24-88)	–	–
S21	Yao^37^	D	2021	China	1	1707	38	57 (23-88)	Req	–
S22	Zhang^38^	D	2020	China	11	3580	42	53 (18-81)	–	–
S23	Zhang^39^	D + S	2022	China	1	260	36	55 (21-94)	–	–
S24	Zhou^40^	D	2020	China	1	974	34	55^c^ (20-97)	–	–
S25	Zhou^41^	D	2020	China	1	894	34	55	–	–
S26	Zhou^42^	D	2021	China	3	602	42	55	–	–
S27	Zhou^43^	D	2022	China	1	818	38	57	Req	–

Abbreviations: FR = female ratio, C = classification, D = detection, S = segmentation, DA = data availability, CA = code availability, Req = available upon request, Com = commercially available.

a From testing dataset.

b Currently not available.

c Median, not average.

All studies used clinical CT scans except one that used PMCT scans.^23^ This study applied specific selection criteria, such as excluding cases of bodies in an advanced state of decomposition or cases of bodies with severe trauma.

Tables 2- 4 contain the average performance metrics obtained by the models in terms of acute rib fracture classification, detection, and segmentation, respectively. Some studies did not report the performance of the model on acute rib fractures alone, but the global performance of the model on acute, healing, and old rib fractures. These cases, which are highlighted in the tables, were excluded from the meta-analysis of acute rib fracture detection.

**Table 2.** Average model performance on acute rib fracture classification.

Study	Dim.	Model architecture	Pretrained	CT scans	ST (mm)	N annot.	Sensitivity (%)	Precision (%)	F1-score (%)
S2	2D	InceptionV3	ImageNet	122	2	498	91	90	–
S6	2D + 3D	ResNet	–	252	5	88	91^a^	69^a^	78^a^
S7	2D	VGG	–	29	1	–	93^a^^b^	89^a^^b^	91^a^^b^

Abbreviations: Dim. = dimensions of the model, N annot. = number of annotations of acute rib fractures.

a Scan-level performance.

b Includes old rib fractures.

**Table 3.** Average model performance on acute rib fracture detection.

Study	Dim.	Model architecture	Pretrained	CT scans	ST (mm)	N annot.	Sensitivity (%)	Precision (%)	F1-score (%)
S1	3D	Faster R-CNN	–	30	3-5	90	82	–	–
S3-1	2D + 3D^a^	Faster R-CNN	ImageNet	80	1-1.25	–	95	90	92
S3-2				55	–	–	97	96	97
S5	3D	U-Net + ResNet	–	123	<1	708	79^b^	43^b^	–
S8	2D	Faster R-CNN + InceptionV2	COCO	19	5	87	71	60	65
S9	3D	U-Net	–	120	1-1.25	882	93	–	–
S10	2.5D	DenseNet + SSD	–	39	0.625	256	65	79	71^c^
S11-1	2D + 3D	CenterNet + ResNet-50 + U-Net	–	1612	0.625-1	9874	93	–	–
S11-2				2319	0.625-1	13524	91	94	92
S12	3D	V-Net	–	350	0.625	1037	91	90	90^c^
S13	3D	V-Net + VGG	–	300	0.625	–	92^b^	95^b^	94^b^
S14	3D	Faster R-CNN	–	56	1-5	199	94	64	76
S15	2D	CenterNet	–	–	5	–	–	89	–
S16-1	3D	U-Net	–	1628	<2	1279	91^b^	–	–
S16-2				1613	<2	3340	85^b^	–	–
S17	2D	U-Net	–	80	1-1.25	–	82	82	82
S18	3D	ResNet + Faster R-CNN	–	510	1.5	688	66^b^	–	–
S19-1	2D + 3D	Faster R-CNN + U-Net	ImageNet	362	0.625-5	1545	84^b^	81^b^	–
S19-2				105	0.625-5	491	85^b^	82^b^	83^b^
S20-1	2D + 3D	CenterNet + U-Net + LSTM	–	120	0.625-1.25	–	92^b^	–	83^b^
S20-2				75	0.625-1.25	–	93^b^	–	81^b^
S21	2D + 3D	U-Net + DenseNet	–	100	<2	436	91	87	89
S22	3D	Foveal + FasterR-CNN	–	198	0.625	865	79^b^	–	–
S23	3D	nnU-Net + DenseNet	–	60	1-1.25	435	95	–	–
S24-1	2D	Faster R-CNN	ImageNet	98	1-5	480	90	85	88
S24-2				33	1	214	95	78	86
S24-3				65	5	266	87	92	90
S24-4				25	1	567	86	81	84
S24-5				25	2	270	83	80	82
S24-6				25	1-2	1073	80	89	84
S25-1	2D + 3D^a^	Faster R-CNN	ImageNet	134	1-2	250	92	83	88
S25-2				62	1	131	97	80	88
S25-3				64	1	144	88	86	87
S26-1	2D + 3D^a^	RetinaNet	–	90	1-1.25	193	91	84	87
S26-2				38	1.5	118	92	83	87
S27	3D	U-Net + attention modules	–	164	1.25-5	–	81	–	–

Abbreviations Dim. = dimensions of the model, N annot. = number of annotations of acute rib fractures.

a Postprocessing.

b Includes healing and old rib fractures.

c Monte Carlo simulated.

**Table 4.** Average model performance on acute rib fracture segmentation.

Study	Dim.	Model architecture	Pretrained	CT scans	ST (mm)	N annot.	Dice (%)	IOU (%)
S4	2D	U-Net	–	50	<2	301	85^a^	80^a^
S9	3D	U-Net	–	120	1-1.25	882	72	56
S17	2D	U-Net	–	80	1-1.25	–	53	–
S23	3D	nnU-Net + DenseNet	–	60	1-1.25	435	63	49

Abbreviations: Dim. = dimensions of the model, N annot. = number of annotations of acute rib fractures, IOU = intersection over union.

a Includes old rib fractures.

### Risk of bias assessment

**Figure 2.**
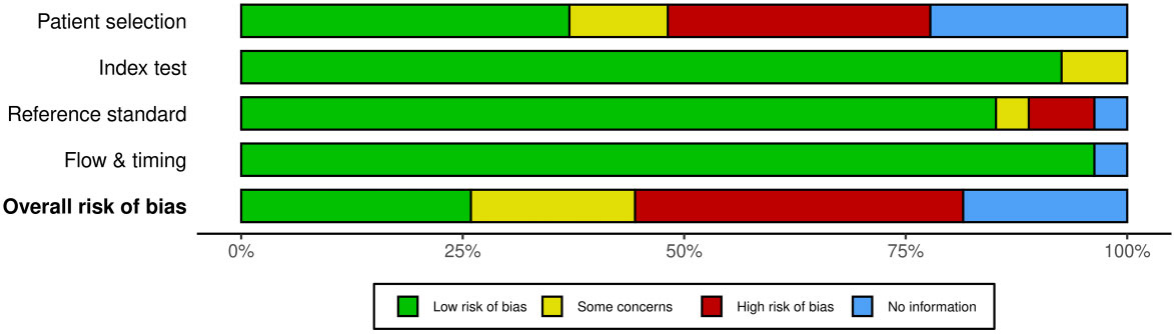
Summary plot of the risk of bias of the studies.

**Figure 3.**
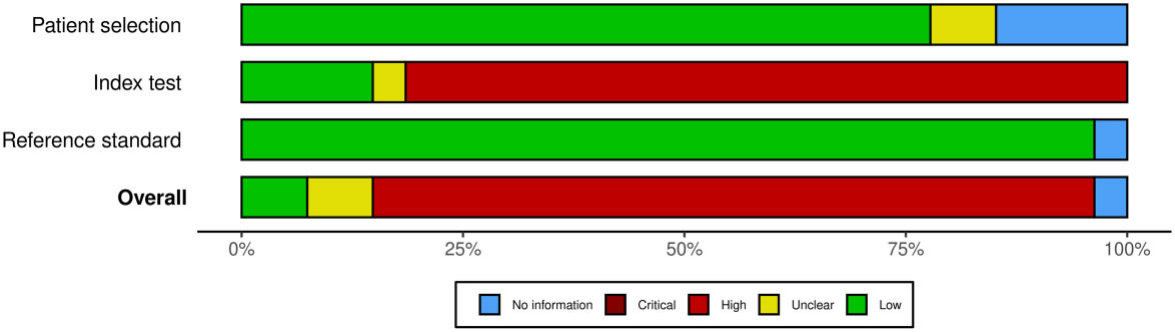
Summary plot of the concerns about applicability of the studies.

Figures 2 and 3 present the summary plots of the ROB and the CAA assessments, respectively.

In the ROB assessment, the patient selection domain was the most affected. About a fourth of the studies did not report the inclusion and exclusion criteria used to select patients. Some studies on rib fracture detection introduced ROB in their reports of precision and F1-score by including control patients in the testing dataset. Additionally, a fourth of the studies collected the CT scans with slice thickness at 5 mm.

For the domain of the index test, there was low ROB in most of the studies, but three quarters of the studies had high CAA, as their models were not publicly available, neither commercially nor as open-source tools.

Concerning the reference standard domain, there was low ROB in the majority of studies, with the exception of one study that used annotations that were not 100% sensitive, and one study in which labels of lesions were removed if they were not annotated by all experts. A model trained with these data may learn to ignore lesions, which would increase the number of FN.

Finally, for the flow and timing domain, no ROB was detected.

The traffic light plots showing the detailed results of the ROB and the CAA assessments can be found in the Supplementary Material.

### Meta-analysis

Only 7 studies were selected for the meta-analysis. As some studies applied their models to more than one testing dataset, the sensitivity meta-analysis consists of 15 points, and the precision and the F1-score meta-analysis consists of 14 points each.

**Figure 4.**
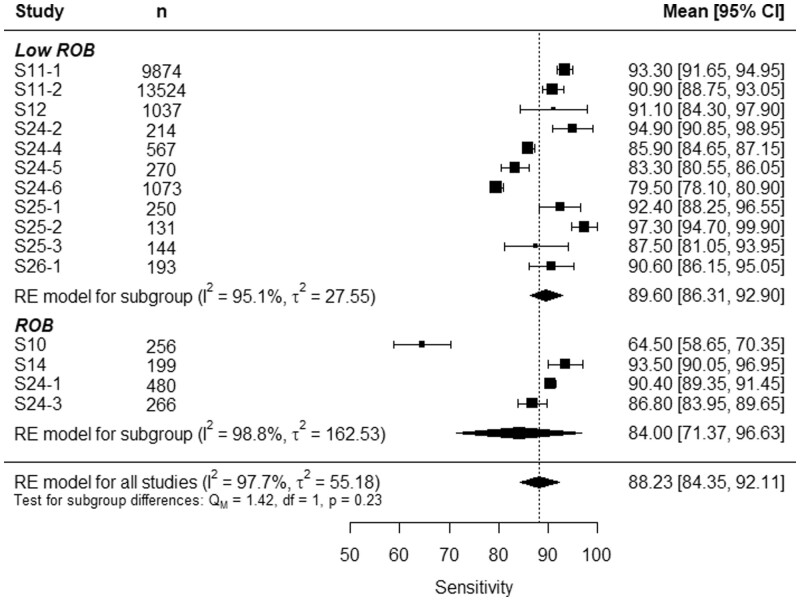
Forest plot of acute rib fracture detection sensitivity. Abbreviations: ROB = risk of bias, RE = random effects.

**Figure 5.**
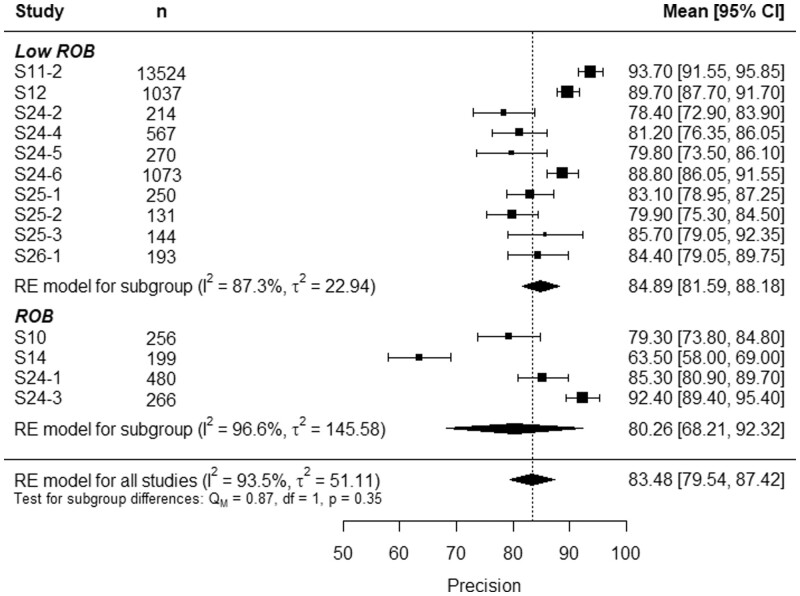
Forest plot of acute rib fracture detection precision. Abbreviations: ROB = risk of bias, RE = random effects.

**Figure 6.**
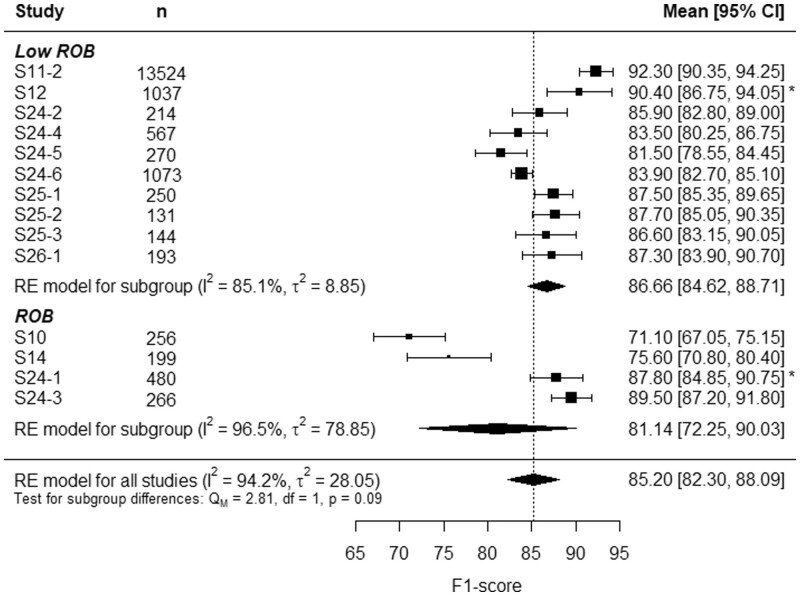
Forest plot of acute rib fracture detection F1-score. Abbreviations: ROB = risk of bias, RE = random effects. *Monte Carlo simulated data.

Figures 4 and 5 show the forest plots of the sensitivity and the precision, respectively. At first glance, one can see that, while models from the low ROB had both high sensitivity and high precision, some studies with ROB had either sensitivity or precision significantly lower than the total RE model average. Indeed, S10 had good precision, but poor sensitivity, and S14 had a notable sensitivity but an improvable precision. This trade-off is no longer observed in Figure 6, where the forest plot of the F1-score studies S10 and S14 from the rest. However, two models from the ROB group, S24-1 and S24-3, presented high sensitivity, precision, and F1-score.

The subgroup analysis led to an averaged sensitivity of 89.60% (95%CI, 86.31%-92.90%) for studies with low ROB and 84.00% (95%CI, 71.37%-96.63%) for studies with ROB. The *I*^2^ statistic was higher than 95% in both subgroups, indicating considerable heterogeneity in both cases. However, the low ROB subgroup was less heterogenic than the ROB subgroup, as the variance *τ*^2^ of the low ROB subgroup was much lower than that of the ROB studies. The subgroup differences test resulted in a *p*-value of 0.23, higher than the threshold of significance 0.1, meaning that there was no evidence that ROB had an impact on rib fracture detection sensitivity.

Similar results were obtained for the precision analysis, where the subgroup of low ROB studies had a precision of 84.89% (95%CI, 81.59%-88.18%), while for the ROB subgroup it was 80.26% (95%CI, 68.21%-92.32%). Again, the low ROB was less heterogenic than the ROB subgroup, with *I*^2^ and *τ*^2^ lower in the low ROB subgroup. The subgroup differences test had a *p*-value of 0.35, above the significance threshold 0.1. Therefore, no evidence was found that ROB had an impact on rib fracture detection precision.

Finally, the F1-score analysis led to the least heterogenic results, with estimates of 86.66% (95%CI, 84.62%-88.71%) and 81.14% (95%CI, 72.25%-90.03%) for the low ROB and the ROB subgroups, respectively. In this case, the subgroup differences test yields a *p*-value lower than 0.1, pointing at the conclusion that ROB has an impact on the F1-score of acute rib fracture detection.

## Discussion and recommendations

Although the treatment of rib fractures is mostly conservative, these lesions are an indicator of associated injuries in more than 90% of patients, and in around 10% of the cases the associated injuries are fatal.^44^ The age of the patient and the number of rib fractures increase the morbidity and mortality of the injuries,^45^^,^^46^ but single rib fractures may also lead to adverse outcomes in 20% of the cases.^47^ By reducing the diagnosis time and achieving a higher sensitivity than radiologists, DL models for rib fracture detection can only improve healthcare.

The selected studies in this systematic review are heterogeneous, with different data and models. The inclusion criteria for patients in each study are also varied. Thus, it is difficult to make any recommendation among the tools presented, as each has its own advantages in a specific application. For instance, while most of the studies focus on acute rib fracture detection, some models can distinguish among acute, healing, and old rib fractures.^18,29,32,36,38^,^40–42^ In other studies, the models can also classify acute rib fractures into displaced, non-displaced, and buckle (or incomplete) rib fractures.^17^^,^^27^^,^^29^^,^^30^^,^^32^^,^^34^^,^^36^^,^^38^^,^^42^ One study analysed the performance of the rib fracture detection model depending on the number of rib fractures in the CT scan.^27^

As a proof of quality of the DL tool, many studies have compared the rib fracture detection performance of the model against that of experienced radiologists. With the assistance of a DL model, the sensitivity of radiologists (60%-80%) can increase up to 20 percentage points while maintaining a similar level of precision (70%-90%) and considerably reducing reading time.^17,18,21^,^24–27^^,29,32,^^35-38^^,^^40-42^

The most common DL model architectures, used by around 10 studies each, are the U-Net^48^ and the Faster R-CNN.^49^ A good example of how rib fracture detection can be resolved via various paths is the use of the U-Net—although this model is designed to perform object segmentation, its results can be postprocessed to output bounding boxes around the predicted object localizations.

There is also considerable heterogeneity concerning the choice of input image dimensions. While the majority of studies decided to extract 3D patches from CT scans to train their models, a number of studies applied 2D models to each individual axial slice. Other researchers extended 2D models to aggregate the results of groups of adjacent slices, which we denote as 2.5D models.

No significant improvement has been found in the performance of a particular choice of architecture and input image dimensions over another. Additionally, we have not observed any significant difference between the performance of models pre-trained on natural image datasets, such as ImageNet^50^ and COCO,^51^ and the performance of the rest of the models.

From the results of the ROB assessment, we recommend that future researchers in this topic make sure to report the patient selection criteria in detail. We believe that the appropriate cohort for a rib fracture detection model is blunt chest trauma patients, that is, patients who are suspected of having rib fractures. In such a cohort, there is no need for a control group of healthy patients (which can lead to a higher number of FP and to an underestimation of precision and F1-score). If patients with healing and old rib fractures are included, such lesions should be annotated accordingly, and the performance of the model should be split into each type of fracture. In addition, we advise not using CT data with 5 mm slice thickness for the training of the models, as such images might blur and hide rib fracture features due to longitudinal partial volume effects.^52^^,^^53^ With the CAA assessment, we remind that the developed DL models should be shared as open-source projects, so the results can be reproduced on different datasets.

The focus of our meta-analysis is acute rib fracture detection, which is the main goal of a rib fracture detection DL model in the emergency department. However, from our point of view, such a model should also have the capacity to distinguish acute from healing and old rib fractures. Otherwise, the model can produce a higher number of FP on patients who had rib fractures previously. Similarly, if the model is trained with CT scans presenting acute, healing, and old rib fractures but only the acute rib fractures are labelled, the model is prone to produce more FN.

The main limitation of our meta-analysis is the reduced number of selected studies, which is a consequence of the fact that the majority of the studies in this systematic review did not report the 95% CI of their results. In particular, the subgroup of studies with ROB had only four points in each forest plot, and in the F1-score forest plot one of the points had to be simulated. In our opinion, performance metrics should be written with their corresponding standard deviations or their 95% CIs.

Finally, it is of particular interest for our team to highlight the opportunities of transfer learning between clinical and PM cases. An advantage of PMCT scans is the absence of imaging artefacts due to breathing and motion of the body. However, PM cases with a high radiological alteration index (RA index)^54^ should be excluded from training datasets. This is because a body with a high RA index presents signs of decomposition, and its CT scan shows air bubbles in many organs and cavities, including the bone marrow. By removing such cases, a properly annotated PMCT dataset can be used to train a DL model for rib fracture detection in a clinical context, and vice versa. We remind that if the goal is to train a DL model for rib fracture detection in patients with suspected blunt chest trauma, such PMCT dataset should only contain PM cases with rib fractures from blunt trauma.

With this systematic review, we have studied DL models for rib fracture classification, detection, and segmentation in CT scans. We have found that many studies do not properly report patient inclusion criteria, and only a few models are available commercially or as open-source tools. Moreover, with our meta-analysis we conclude that low ROB studies have significantly better performance in acute rib fracture detection with DL models.

## Supplementary Material

tqae014_Supplementary_Data
