# Supplementary material for: Deep learning for acute rib fracture detection in CT data: a systematic review and meta-analysis
Source: Br J Radiol. 2024 Jan 13;97(1155):535–43. doi: 10.1093/bjr/tqae014 (PMC11027249; doi:10.1093/bjr/tqae014)
Supplement: tqae014_Supplementary_Data [file tqae014_supplementary_data.docx]

1. **Formulae of performance metrics**

The performance metrics we decided to extract for rib fracture detection studies were the sensitivity, precision and F1-score. The first two can be defined as

$$Sens= \frac{TP}{TP+FN} , Prec= \frac{TP}{TP+FP}$$

where TP stands for true positives, FP for false positives, and FN for false negatives, all of them at lesion-level. The F1-score metric can be defined as the harmonic mean of these metrics, that is

$$F1= 2 \frac{Sens\cdot Prec}{Sens+Prec} .$$

For rib fracture segmentation studies, the Dice score and Intersection Over Union (IOU) were extracted. These can be defined as

$$Dice= \frac{2 TP}{2 TP+FP+FN} , IOU= \frac{TP}{TP+FP+FN}$$

where TP, FP and FN here are at pixel-level.

1. **Risk of bias and concerns about applicability signalling questions**

Below is the list of signalling questions used to assess the risk of bias (ROB) of the selected studies.

- Patient selection
  - *Was a consecutive or random sample of patients enrolled?*

Most studies use consecutive or random samples of patients. Only studies with a considerably low number of patients selected in a long period of time are suspicious of introducing ROB in this domain.

- - *Was a case–control design avoided?*

In rib fracture detection or segmentation studies, there is no need of a control group, as chest trauma patients always present healthy ribs. Including a control group produces an underestimation of metrics such as precision or F1-score, as it can generate more FP. Only if a model performs CT scan classification into healthy patients and patients with rib fractures is justified to have a control group.

- - *Did the study avoid inappropriate exclusions?*

Studies with specific exclusion criteria, such as dropping CT scans that were not preprocessed successfully, or studies that do not apply standard exclusion criteria, such as excluding CT scans with breathing artifacts, are suspicious of introducing ROB in this domain. In addition, studies using CT scans with slice thickness of 5 mm are also at risk of introducing bias to their results, as some rib fractures might be hidden due to longitudinal partial volume effects.

- Index test
  - (Blinding) *Were the index test results interpreted without knowledge of the results of the reference standard?*

No studies introduce ROB via this signalling question.

- - *If a threshold was used, was it prespecified?*

Some studies define hyperparameters after applying the model on the test set. Such studies are considered to introduce ROB in this domain.

- Reference standard
  - (100% sensitive reference standard) *Is the reference standard likely to correctly classify the target condition?*

We assess high ROB for studies that report that their annotations are incomplete due to disagreements among radiologists, or studies that report that the DL tool detected some rib fractures that were not part of the ground truth.

- - (Blinding) *Were the reference standard results interpreted without knowledge of the results of the index test?*

One study introduces ROB because it uses a reference standard that reached their final decision after checking the results of the DL tool.

- Flow and timing
  - **REMOVED.** *Was there an appropriate interval between the index test and reference standard?*

This signalling question is not applicable in this systematic review, as both the radiologists and the DL tool analyse the very same CT scan. The interval between reference standard and index test is 0 in all cases.

- - *Did all patients receive the same reference standard?*

Only one study does not report information concerning the reference standard to all patients in the training and testing datasets, introducing ROB.

- - *Were all patients included in the analysis?*

No studies introduce ROB via this signalling question.

As for the concerns about applicability (CAA), the signalling questions used have been the following.

- Patient selection
  - Are There Concerns That the Included Patients and Setting Do Not Match the Review Question?

Studies with unusual or unreported patient selection criteria introduce CAA.

- Index test
  - *Are There Concerns That the Index Test, Its Conduct, or Its Interpretation Differ From the Review Question?*

In particular for this systematic review, as it concerns deep learning models, the main concern about applicability of a tool introduced by a study is the fact that the tool is not publicly available. Thus, studies presenting models that are not commercially available or that are not open-source are considered to have high CAA in this domain.

- Reference standard
  - *Are There Concerns That the Target Condition as Defined by the Reference Standard Does Not Match the Question?*

Only in one study, the reference standard is not defined, which introduces CAA.

1. **Additional figures and tables**

Below are some additional figures and tables that complement some information of our systematic review. Figures 1 and 2 show the traffic light plots of the ROB and CAA assessments, that is, the results in each domain for each study, and the overall for each study. Table 1 gathers extra information about the studies, like their title, patient selection criteria and whether the CT scans include cases with healing and old rib fractures.

It is worth mentioning that in Figure 1 S24 is marked as having high ROB since two of its models, S24-1 and S24-3, are trained with 5 mm slice thickness CT scans. However, the rest of its models, namely S24-2, S24-4, S24-5 and S24-6, do not present ROB.


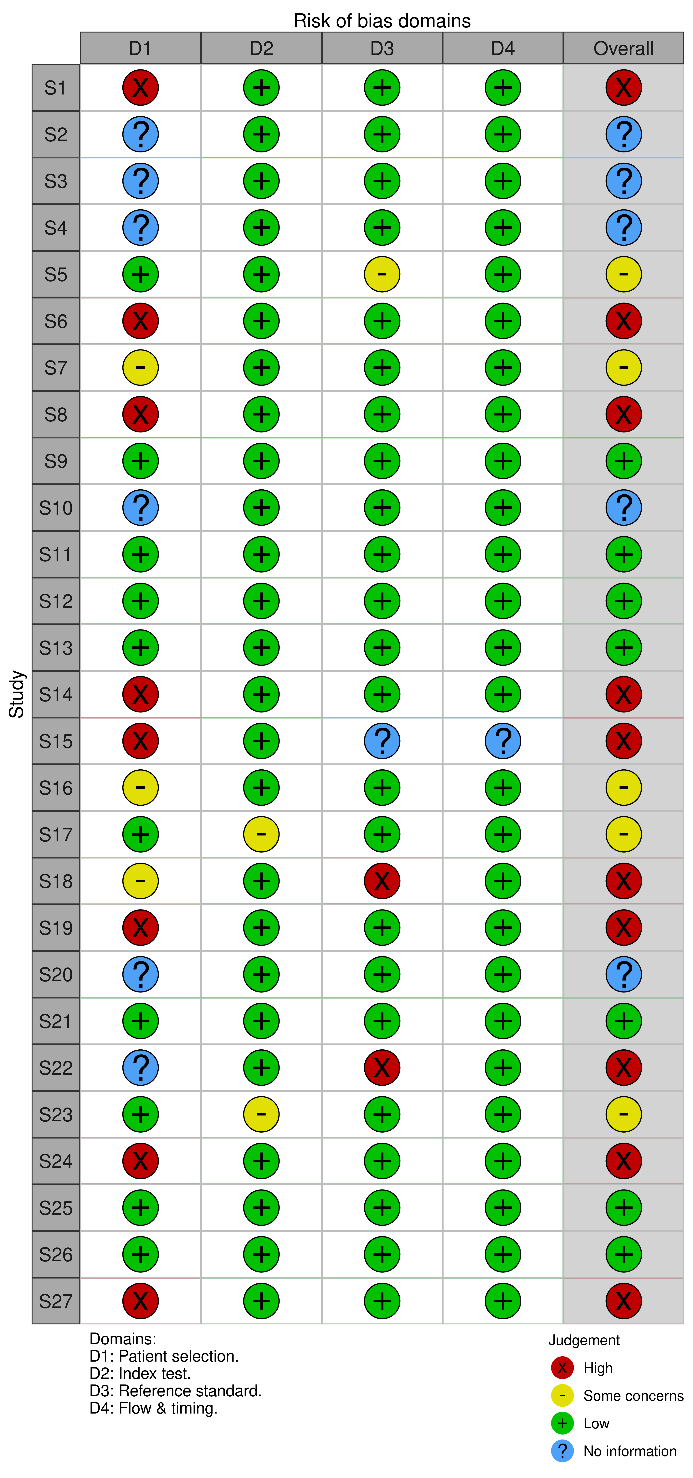


Figure 1: Traffic light plot of the risk of bias of the studies.


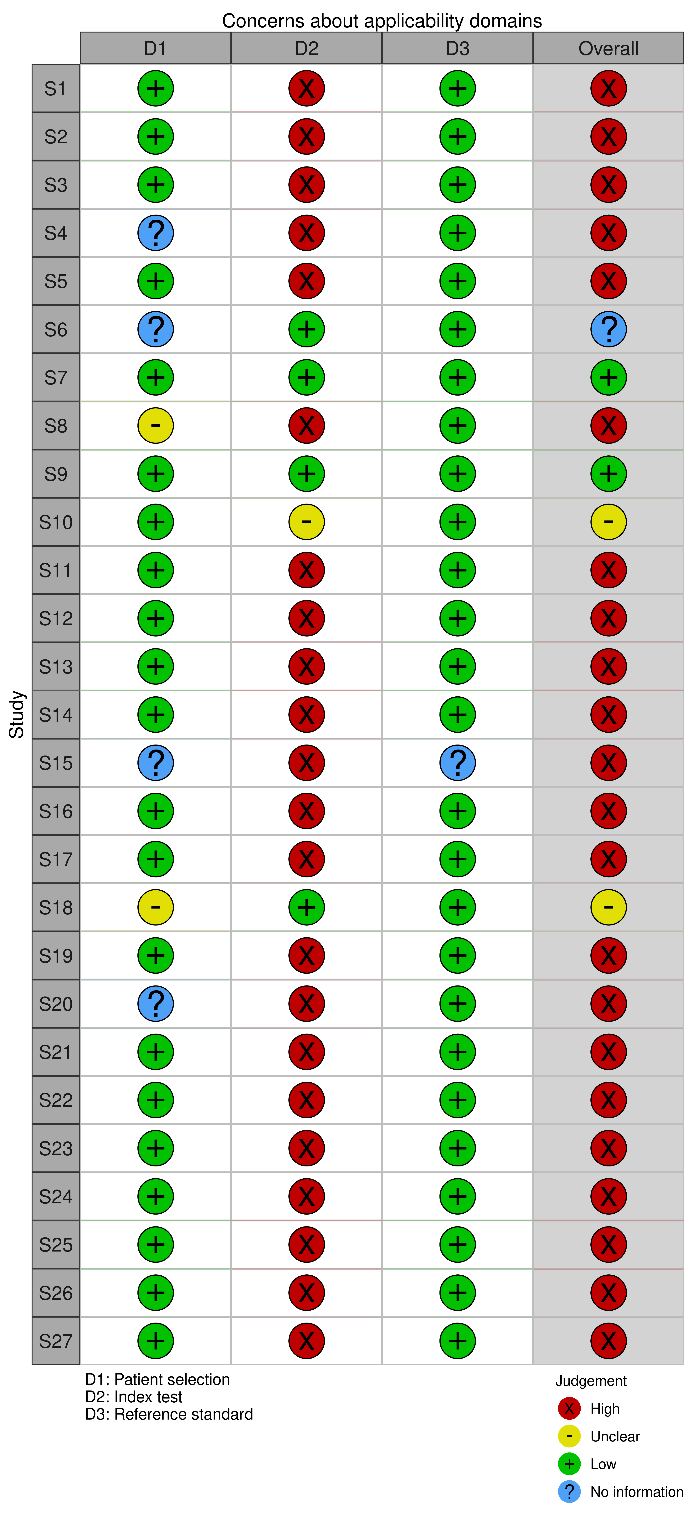


Figure 2: Traffic light plot of the concerns about applicability of the studies.

Table 1. Inclusion criteria for patients.

| Study | Title | Inclusion criteria | Healing and old rib fractures |
| --- | --- | --- | --- |
| S1 | Detection of acute rib fractures on CT images with convolutional neural networks: effect of location and type of fracture and reader's experience | Patients with trauma | - |
| S2 | Assessing the speed-accuracy trade-offs of popular convolutional neural networks for single-crop rib fracture classification | Patients with and without rib fractures | Yes |
| S3 | FasterRib: A Deep Learning Algorithm to Automate Identification and Characterization of Rib Fractures on Chest Computed Tomography Scans | (RibFrac subset) | - |
| S4 | Deep learning-based framework for segmentation of multiclass rib fractures in CT utilizing a multi-angle projection network | Patients with rib fractures | Yes |
| S5 | Comparison and verification of two deep learning models for the detection of chest CT rib fractures | Patients with chest trauma | Yes |
| S6 | Slice grouping and aggregation network for auxiliary diagnosis of rib fractures | Patients with and without rib fractures | - |
| S7 | RiFNet: Automated rib fracture detection in postmortem computed tomography | PM cases without signs of advanced decomposition and without extensive damage to the corpse | Yes |
| S8 | Automated fracture screening using an object detection algorithm on whole-body trauma computed tomography | Patients with pelvic, rib or spine fractures | - |
| S9 | Deep-learning-assisted detection and segmentation of rib fractures from CT scans: Development and validation of FracNet | (RibFrac) Patients with rib fractures | - |
| S10 | Rib fracture detection in computed tomography images using deep convolutional neural networks | Patients with chest injuries | - |
| S11 | An Automatic Fresh Rib Fracture Detection and Positioning System Using Deep Learning | Patients with chest trauma | - |
| S12 | Deep learning-based computed tomography applied to the diagnosis of rib fractures | Patients with chest trauma | - |
| S13 | A fully automated rib fracture detection system on chest CT images and its impact on radiologist performance | Patients with chest trauma | Yes |
| S14 | Development of an artificial intelligence-assisted computed tomography diagnosis technology for rib fracture and evaluation of its clinical usefulness | Patients with rib fractures and control cases | - |
| S15 | Rib fracture detection in chest CT image based on a centernet network with heatmap pyramid structure | Patients with chest CT scans | - |
| S16 | Assessment of automatic rib fracture detection on chest CT using a deep learning algorithm | Patients with trauma | Yes |
| S17 | Composite Attention Residual U-Net for Rib Fracture Detection | (RibFrac subset) | - |
| S18 | Assessment of a Deep Learning Algorithm for the Detection of Rib Fractures on Whole-Body Trauma Computed Tomography | Patients with trauma | Yes |
| S19 | Development and Evaluation of a Deep Learning Algorithm for Rib Segmentation and Fracture Detection from Multicenter Chest CT Images | Patients with rib fractures and control cases | Yes |
| S20 | Development and assessment of deep learning system for the location and classification of rib fractures via computed tomography | Patients with rib fractures | Yes |
| S21 | Rib fracture detection system based on deep learning | Patients with rib fractures | - |
| S22 | Improving rib fracture detection accuracy and reading efficiency with deep learning-based detection software: a clinical evaluation | Patients with blunt chest trauma | Yes |
| S23 | An Algorithm for Automatic Rib Fracture Recognition Combined with nnU-Net and DenseNet | (RibFrac subset) | - |
| S24 | Automatic Detection and Classification of Rib Fractures on Thoracic CT Using Convolutional Neural Network: Accuracy and Feasibility | Patients with rib fractures | Yes |
| S25 | Automatic detection and classification of rib fractures based on patients' CT images and clinical information via convolutional neural network | Patients with rib fractures | Yes |
| S26 | Precise anatomical localization and classification of rib fractures on CT using a convolutional neural network | Patients with rib fractures | Yes |
| S27 | Rib Fracture Detection with Dual-Attention Enhanced U-Net | Patients with chest trauma | - |
